# Erratum to “With Appreciation” [Advances in Nutrition, Volume 14, Issue 1, January 2023, Pages 211–213]

**DOI:** 10.1016/j.advnut.2023.02.001

**Published:** 2023-02-21

We thank the following individuals for taking the time necessary to evaluate original manuscripts. Anonymous, conscientious, fair, and timely peer review is the lifeblood of the journal.

**Table:** 

Esther Aarts
Enass A. Abdel-Hameed
Rachel Loran Adams
Kanika Agarwal
Prekshi Aggarwal
Noha Ahmed Nasef
Rand Talal Akasheh
Janice Lee Albert
Celeste Alexander
Lindsay H. Allen
Mandana Amir Shaghaghi
Elitsa Ananieva
Anitha Ananthan
Alex K. Anderson
Tracy Anthony
John W. Apolzan
Patrice Armstrong
Joanne E. Arsenault
Diana V. Artene
Maryam Asadi
Gayatri Athalye-Jape
Domenico Azzolino

Jiyoung Bae
David Baer
Connie Bales
Janett Barbaresko
Kathryn Barger
Jennifer L. Barnes
Maria Barrero
Anthony J. Basile
Marlana Bates
Jo-Anna Baxter
Megan R. Beggs
Tair Ben-Porat
Andrew Berardy
Silvia Berciano
Bronwyn Sarah Berthon
Charles Bevins
Julia Bird
Elisabet Borsheim
Roger Bouillon
Shanthy Bowman
Anne Lise Brantsæter
Lauren Roukia Brink
Inge Brouwer
Rachel Brown
Polina Bugaeva
James Butcher
Michael Butler

Yasemin Çakir Gökkurt
Vittorio Calabrese
Mona S. Calvo
Mary Ellen Camire
Chang Cao
Chao Cao
Kelly Copeland Cara
Franck Carbonero
Susan Carlson
Kellie O. Casavale
Fatma Cebeci
Catherine Chan
Jorge E. Chavarro
Oliver Chen
Junrui Cheng
Katherine E. Chetta
Clara E. Cho
Mei Chung
Soonkyu Chung
Mei Chung
Catharine Clark
Zachary S. Clayton
Rodolfo Castilho Clemente
Esi Komeley Colecraft
Lorraine S. Cordeiro
Dolores Corella
Maria Magel Costa
Rebecca B. Costello
Joshua Costin

Wendy Dahl
Zhaoli Dai
Omar Dary
Hassan S. Dashti
Alberto Davalos
Harry Dale Dawson
Alie de Boer
Kees de Graaf
Lisette de Groot
Vicky De Preter
Baukje de Roos
Jeanne de Vries
Treena Delormier
Felipe Mendes Delpino
Flore Depeint
Jaapna Dhillon
Sara C. Di Rienzi
Jutta Dierkes
Loretta DiFrancesco
Olupathage Chandani Dinesh
Marlou L. Dirks
Sharon Donovan
James Drackley
Jean-Philippe Drouin-Chartier
Mengxi Du
Valerie Duffy
Courage Sedem Dzah

Brendan Egan
Manfred Eggersdorfer
Aristides Eliopoulos
Nancy J. Emenaker
Laurel English
John Erdman
Abdulkerim Eroglu

Susan Fairweather-Tait
Gary Lynn Ferguson
Mackenzie Ferrante
Mario Ferruzzi
Edith Feskens
Martha S. Field
Roger Fielding
John Finley
Sheila Fleischhacker
Michael Flock
Leigh A. Frame
Craig Friesen
Simonetta Friso
Edward A. Frongillo
付星 Fu

Jose J. Gaforio
Jaime J. Gahche
Daniel D. Gallaher
Monica Gallo
Kan Gao
Xiang Gao
Jessica L. Garay
Christopher D. Gardner
Dawd Gashu
Nori Geary
Nirjhar Ruth Ghosh
Vincenza Gianfredi
Michael J. Gibney
Rachel Gibson
Stephanie Pritchard Gilley
Andrea Glenn
Stephanie Goldstein
Manuel Gonzalez Ronquillo
Gijs Goossens
Sarah Grafenauer
Frank Greer
Jessica A. Grieger
Inge Groenendijk
Vivienne Guan
Patricia Guenther
Charlotte Gupta

Hajo Haase
Megan L. Hammersley
John Harwood
Danielle Haslam
Mark Haub
Anna Hayes
James R. Hebert
Erin Henderson
Linda Milou Hengeveld
Diego Hernandez-Saavedra
Julie M. Hess
David Hill
Kendal Hirschi
Emily Ho
Denise K. Houston
Debbie Humphries
Dana Hunnes

Fumiaki Imamura
Suvi T. Itkonen

David Jacobs
Paul F. Jacques
Supriya Jagga
Brittany James
Thomas Jansson
Sookyoung Jeon
Xiangming Ji
Qingkui Jiang
Rulan Jiang
Xinyin Jiang
Tricia J. Johnson
Donald Jump

Fawzi Kadi
Stanley George Kailis
Kathryn Kaiser
Anastasia Kanellou
J. Philip Karl
David L. Katz
Maryam Kebbe
Nicole J. Kellow
Linsay Ketelings
Daniel Kirk
Sharon Kirkpatrick
Nicole Kiss
Tonny Kiyimba
Kevin Klatt
Jochen Klein
Ronald E. Kleinman
David M. Klurfeld
衣美 Kondo
Kristine G. Koski
Sibylle Kranz

Chao-Qiang Lai
Nemanja Lakicevic
Kelly Lambert
Bobbi Langkamp-Henken
Olaf Larsen
Kevin D. Laugero
Christine Leroux
Jia Li
Paul Lips
Erikka Loftfield
Sofia Lotti
Nicola Lowe
Jing Lu

David Ma
Jiantao Ma
Joreintje Mackenbach
Karen L. Madsen
Kevin Maki
Kelsey McKee Mangano
Stephen A. Maris
Richard Mattes
Christophe Matthys
James McClung
Krista McCoy
Megan A. McCrory
Robin McKinnon
Alexander McLain
David Mela
Jordi Merino
John Jeshurun Michael
Joshua Douglas Miller
Marshall Miller
Brandy-Joe Milliron
Anne Marie Minihane
Thais Miola
Marybeth Mitcham
Diane C. Mitchell
Taulant Muka
Daniel Munblit

Muzi Na
Perrine Nadaud
Ravinder Nagpal
Nirmala Nanguneri
Nenad Naumovski
Elizabeth P. Neale
Julie Elyse Nevins
Qin Xiang Ng
Daiva Nielsen
Sabrina E. Noel
Eric B. Nonnecke

Julie Obbagy
Kimberly O'Brien
Caitlin O'Connell
Lauren Elizabeth O'Connor
Sydney G. O'Connor
Rudy Ortiz
Alice Owen
Asil Oztekin

Christopher Papandreou
Yong-Moon Park
Misha Patel
Alex Pepetone
Carmen M. Perez
Kristina S. Petersen
Brian Piccolo
Matthew Picklo
Collin Popp

Ramkripa Raghavan
Kathleen Ragsdale
Sujatha Rajaram
Robert A. Rastall
Elham Razmpoosh
Natalia Rebolledo
R. Scott Rector
Caroline Richard
Rene Rizzoli
Joseph L. Roberts
Nancy Rodriguez
Carmen Rodríguez-García
Brian Ross
Robert B. Rucker
Cherie Ann Russell

Priya S
Joan Sabate
Miguel Saenz de Pipaon Marcos
Maryam Saghafi-Asl
Nadine Sahyoun
Aleix Sala-Vila
Amin Salehi-Abargouei
Abril Iliana Sánchez-Rosales
Peter Sarich
Caleigh Sawicki
Sabrina Schlesinger
Lukas Schwingshackl
Jonathon Senefeld
Katelyn E. Senkus
Barbara Shukitt-Hale
Gina Segovia Siapco
Joanne Slavin
Tomasz Śledziski
A. David Smith
Elliot O'Brian Smith
Taryn J. Smith
Patrick Solverson
John Sorkin
Thalia Morrow Sparling
J. David Spence
Wilma Steegenga
Charles B. Stephensen
Barbara Stoecker
Brianna Stubbs
April Stull
Xiong Su
Padhmanand Sudhakar
Simon Siu-Man Sum
Edyta Szczerba

Yumie Takata
Minghua Tang
Sherry A. Tanumihardjo
Toby Tao
Emir Tas
Elisabeth Temme
Simon N. Thornton
Daniel Tomé
Maret G. Traber
Daniel Traylor
Igor Alfonso Trujillo
Chandler Tucker
Claire Nora Tugault-Lafleur
Nancy Turner
Bradley Turnwald

Iris van Damme
Ondine van de Rest
Linda Van Horn
Stephan van Vliet
Arthur R.H. van Zanten
David Vauzour
Ramon Velazquez
Harm Veling
Sudha Venkataraman
Nalleli Vivanco Karlsson
Trudy Voortman
Alina Vrieling

Taylor C. Wallace
Ye Wang
Robert Ward
Connie Weaver
Jean Welsh
Michael Wenger
Evertine Wesselink
Jay Whelan
Corrie Whisner
Parke Wilde
Alexei Wong
Chi Wut Wong
Hao Wu
Xianli Wu

Hidetoshi Yamada
Brandon Yates
Alison Yaxley
Sonja Yokum
Tri Yuliani

Ioannis Zabetakis
Janos Zempleni
Liangzi Zhang
Tianou Zhang
Ju-Sheng Zheng
Pan Zhuang
Angela M. Zivkovic

